# Convergent evidence from systematic analysis of GWAS revealed genetic basis of esophageal cancer

**DOI:** 10.18632/oncotarget.10133

**Published:** 2016-06-17

**Authors:** Xue-xin Gao, Lei Gao, Jiu-qiang Wang, Su-su Qu, Yue Qu, Hong-lei Sun, Si-dang Liu, Ying-li Shang

**Affiliations:** ^1^ Department of Thoracic Surgery, Central Hospital of Tai'an, Tai'an, Shandong, China; ^2^ Department of Shandong Provincial Research Center for Bioinformatic Engineering and Technique, School of Life Sciences, Shandong University of Technology, Zibo, Shandong, China; ^3^ Department of State Key Laboratory of Membrane Biology, Institute of Zoology, Chinese Academy of Sciences, Beijing, China; ^4^ Department of Key Laboratory of Mental Health, Institute of Psychology, Chinese Academy of Sciences, Beijing, China University of Chinese Academy of Sciences, Beijing, China; ^5^ Department of Pathology, University of Texas, Medical Branch, Galveston, Texas, USA; ^6^ Department of Key Laboratory of Animal Epidemiology and Zoonosis, Ministry of Agriculture, College of Veterinary Medicine and State Key Laboratory of Agrobiotechnology, China Agricultural University, Beijing, China; ^7^ Department of Preventive Veterinary Medicine, College of Animal Science and Veterinary Medicine, Shandong Agricultural University, Tai'an, Shandong, China

**Keywords:** GWAS, genetic basis, pathway, network, esophageal cancer

## Abstract

Recent genome-wide association studies (GWAS) have identified single nucleotide polymorphisms (SNPs) associated with risk of esophageal cancer (EC). However, investigation of genetic basis from the perspective of systematic biology and integrative genomics remains scarce.

In this study, we explored genetic basis of EC based on GWAS data and implemented a series of bioinformatics methods including functional annotation, expression quantitative trait loci (eQTL) analysis, pathway enrichment analysis and pathway grouped network analysis.

Two hundred and thirteen risk SNPs were identified, in which 44 SNPs were found to have significantly differential gene expression in esophageal tissues by eQTL analysis. By pathway enrichment analysis, 170 risk genes mapped by risk SNPs were enriched into 38 significant GO terms and 17 significant KEGG pathways, which were significantly grouped into 9 sub-networks by pathway grouped network analysis. The 9 groups of interconnected pathways were mainly involved with muscle cell proliferation, cellular response to interleukin-6, cell adhesion molecules, and ethanol oxidation, which might participate in the development of EC.

Our findings provide genetic evidence and new insight for exploring the molecular mechanisms of EC.

## INTRODUCTION

Esophageal cancer is the 6^th^ leading cause of death from cancer and the 8^th^ most common cancer in the world [1]. Epidemiological researches have demonstrated that both environmental factors (eg. alcohol consumption) and genetic factors (genetic variants) contribute to the risk of EC development [2]. Meanwhile, genome-wide association study (GWAS) offers the opportunity to investigate genetic factors involved in this complex disorder and several single nucleotide polymorphisms (SNPs) have been identified to be significantly associated with risk of EC [3]. However, results from current GWAS of EC mainly focus on individual SNPs with highly statistical significance (P-value < 5.0E-08), investigation of genetic basis from the perspective of systematic biology and integrative genomics remains scarce.

Due to the polygenic risk of complex disorders, the effect size attributable to individual genetic variants was typically modest, suggesting that individual genetic variants identified by GWAS may only accounted for a very small amount of the genetic risk and heritability of complex disorders [4]. The combined effect of multi genetic variants or genes with modest effect also plays important roles in genetic basis of complex disorders such as esophageal cancer [5]. GWAS provides us an important data source for the investigation of multi-variant/gene effect. Moreover, combining GWAS data with bioinformatics methods such as expression quantitative trait loci (eQTL) analysis, pathway based analysis, and network analysis, the integrative genomics approach could provide systematic evidence to genetic basis of disease [6].

In this study, we explored genetic basis of EC by comprehensive data mining and systematical data analysis based on GWAS data and a series of bioinformatics methods, which may provide better understanding for the molecular mechanisms that contribute to the development of EC.

## RESULTS

### Identification of SNPs associated with risk of esophageal cancer

By comprehensive data search and collection, we obtained a total of 7 published GWAS of esophageal cancer [21]-[27], in which the sample size ranged from four thousands to twenty thousands and the ethnic groups of samples were mainly Asian descent except one study with European descent, detecting 500 thousands to one million of SNPs from the whole genome in each GWAS. A total of 211 SNPs reported with P-value <5.0E-05 were obtained and considered as risk SNPs of esophageal cancer. Summary of GWAS including disease, ethnic groups, sample size, genotyping platform, and number of detected SNPs was shown in Table 1 and details of reported SNPs, their P-values and odds ratios were shown in Supplementary Table S1. As shown in Supplementary Table S5, results of power analysis demonstrated for two-stage designed GWAS, in the initial analysis, studies with initial sample size larger than 3000 had more than 90% power of detecting risk SNPs. While initial sample size of study [27] was 1109, the power of detecting risk SNPs with allele frequency of 0.1 and 0.9 was less than 70%. When combining initial sample and replicated sample, all studies achieved more than 90% power at any allele frequency level.

**Table 1:** Summary of esophageal cancer GWAS

Study	Disease	Ethnic groups	Initial sample size (case/control)	Replicated Sample size (case/control)	Genotyping platform	No. of detected SNPs	No. of reported SNPs with P < 5.0E-05
Levine DM [21]	EAC	European	1,516/3,209	874/6,911	Illumina	922,031	13
Jin G [22]	Multiple cancers (including ESCC)*	Asian (Chinese)	2,031/4,006	3,006/11,436	Affymetrix	NA	1
Wu C [23]	ESCC	Asian (Chinese)	2,031/2,044	8,092/8,620	Affymetrix	666,141	151
Wu C [24]	ESCC	Asian (Chinese)	2,031/2,044	3,986/4,157	Affymetrix	666,141	11
Abnet CC [25]	ESCC and gastric cancer*	Asian (Chinese)	1,898/2,100	NA	Illumina	551,152	7
Wang LD [26]	ESCC	Asian (Chinese)	1,077/1,733	7,673/11,013(Han Chinese), 303/537(Uygur-Kazakh Chinese)	Illumina	506,666	18
Cui R [27]	ESCC	Asian (Japanese)	182/927	782/1,898	Illumina	359,195	12

EAC: esophageal adenocarcinoma; ESCC: esophageal squamous cell carcinoma; NA: not applicable.

* Only SNPs associated with ESCC were included in this study.

### Functional annotation and expression quantitative trait loci (eQTL) analysis

As shown in Supplementary Table S2, for 211 risk SNPs, related chromosome, genome position, allele change, and mapped gene/region were annotated. These 211 risk SNPs were mapped into 170 genes, which were considered as genes associated with risk of esophageal cancer. By eQTL analysis, among 211 risk SNPs, we observed 44 SNPs with significant gene expression changes in several esophageal tissues, including esophagus muscularis (sample size: 241), esophagus mucosa (sample size: 218), esophagus gastroesophageal junction (sample size: 235), with permutation adjusted P–values < 0.05. Detailed results including SNPs, esophageal tissues, gene with altered expressions and P-values were displayed in Supplementary Table S3.

### Pathway enrichment analysis

By pathway enrichment analysis, with the threshold of Benjamini-adjusted P-value < 0.05, we obtained 38 significant GO terms and 17 significant KEGG pathways, which were considered as significant pathways of esophageal cancer. Meanwhile, fold enrichment of risk genes in each significant pathway were all larger than 1.5, demonstrating risk genes were significantly enriched in these pathways. The details of significant pathways including pathway ID, P-values, involved genes, and fold enrichment were shown in Table 2.

**Table 2:** Significant pathways of esophageal cancer

Pathway ID	Pathway title	Adjusted P-value*	Associated Risk Genes Found	% Associated Risk Genes	Fold enrichment
GO:0002377	immunoglobulin production	6.79E-03	FOXP1, IL6, XBP1, XRCC4	4.44	7.83
GO:0002700	regulation of production of molecular mediator of immune response	7.54E-03	FOXP1, IL6, TGFB2, XBP1	4.26	7.50
GO:0004022	alcohol dehydrogenase (NAD) activity	1.50E-10	ADH1A, ADH1B, ADH1C, ADH4, ADH6, ADH7	75.00	132.18
GO:0004030	aldehyde dehydrogenase (NAD(P)+) activity	1.08E-04	ADH4, ADH7, ALDH2	37.50	66.09
GO:0005178	integrin binding	3.50E-03	ADAMTS5, FN1, ITGA6, PPAP2B, PTPN2	4.35	7.66
GO:0005501	retinoid binding	6.21E-03	ADH4, ADH7, UGT2B7	6.82	12.02
GO:0006069	ethanol oxidation	2.66E-11	ADH1A, ADH1B, ADH1C, ADH4, ADH6, ADH7, ALDH2	63.64	112.15
GO:0006493	protein O-linked glycosylation	8.74E-03	ADAMTS5, GALNT13, MUC4, ST6GAL1	4.00	7.05
GO:0006656	phosphatidylcholine biosynthetic process	3.32E-03	CHEK2, FABP5, SLC44A5	9.38	16.52
GO:0006805	xenobiotic metabolic process	3.34E-04	ADH1A, ADH1B, ADH1C, ADH4, ADH6, ADH7, ALDH2, SULT1A1	4.40	7.75
GO:0007431	salivary gland development	1.22E-03	FGFR2, IL6, TGFB2, XBP1	8.70	15.32
GO:0010883	regulation of lipid storage	5.73E-03	IL6, PTPN2, SREBF2	7.14	12.59
GO:0030134	ER to Golgi transport vesicle	2.11E-04	HLA-A, HLA-DPA1, HLA-G, KIAA0368, SREBF2	9.62	16.95
GO:0030176	integral component of endoplasmic reticulum membrane	2.05E-04	CLN3, HLA-A, HLA-DPA1, HLA-G, SREBF2, TBL2, XBP1	5.65	9.95
GO:0030818	negative regulation of cAMP biosynthetic process	4.25E-03	EDNRA, GNAI2, GRM3	8.33	14.69
GO:0031016	pancreas development	7.75E-03	GATA6, GNAI2, IL6, XBP1	4.17	7.34
GO:0031069	hair follicle morphogenesis	3.32E-03	FGFR2, RUNX1, TGFB2	9.38	16.52
GO:0032729	positive regulation of interferon-gamma production	2.19E-03	HLA-A, HLA-DPA1, IL18R1, PDE4D	7.02	12.37
GO:0033002	muscle cell proliferation	3.53E-04	EDNRA, FGFR2, FOXP1, GATA6, IL6, PDE4D, TGFB2	5.07	8.94
GO:0034774	secretory granule lumen	3.71E-03	FN1, GNAI2, IL6, TGFB2	5.63	9.93
GO:0042093	T-helper cell differentiation	5.42E-03	FOXP1, IL18R1, IL6	7.50	13.22
GO:0042307	positive regulation of protein import into nucleus	2.01E-03	IL18R1, IL6, KANK1, XBP1, ZIC1	5.32	9.37
GO:0042439	ethanolamine-containing compound metabolic process	7.43E-03	CHEK2, CLN3, FABP5, SLC44A5	4.30	7.58
GO:0042987	amyloid precursor protein catabolic process	1.42E-03	CLN3, FKBP1A, HAP1	14.29	25.18
GO:0043368	positive T cell selection	2.16E-03	DOCK2, IL6, PTPN2	11.54	20.33
GO:0046164	alcohol catabolic process	1.17E-02	ADH4, ADH7, ALDH2	5.08	8.96
GO:0046631	alpha-beta T cell activation	3.06E-03	DOCK2, FOXP1, HLA-A, IL18R1, IL6	4.59	8.08
GO:0048634	regulation of muscle organ development	2.68E-03	BDNF, FGFR2, FOXP1, GATA6, IL6	4.81	8.47
GO:0048659	smooth muscle cell proliferation	1.91E-03	EDNRA, FGFR2, FOXP1, IL6, PDE4D	5.43	9.58
GO:0050839	cell adhesion molecule binding	7.84E-05	ADAMTS5, FN1, ITGA6, NRXN1, POSTN, PPAP2B, PTPN2, PTPRM, TRPC4	4.84	8.53
GO:0051965	positive regulation of synapse assembly	2.16E-03	BDNF, CBLN1, NRXN1	11.54	20.33
GO:0055025	positive regulation of cardiac muscle tissue development	2.77E-03	FGFR2, FOXP1, GATA6	10.34	18.23
GO:0060038	cardiac muscle cell proliferation	9.07E-04	FGFR2, FOXP1, GATA6, TGFB2	9.52	16.78
GO:0060337	type I interferon signaling pathway	5.32E-03	HLA-A, HLA-G, PTPN2, USP18	4.94	8.70
GO:0071354	cellular response to interleukin-6	2.09E-03	IL6, PHB, PTPN2	12.00	21.15
GO:0071556	integral component of lumenal side of endoplasmic reticulum membrane	2.77E-03	HLA-A, HLA-DPA1, HLA-G	10.34	18.23
GO:1901019	regulation of calcium ion transmembrane transporter activity	1.42E-02	FKBP1A, HAP1, PDE4D	4.69	8.26
GO:1902106	negative regulation of leukocyte differentiation	5.32E-03	HLA-G, PTPN2, RUNX1, THOC5	4.94	8.70
KEGG:00010	Glycolysis / Gluconeogenesis	6.91E-06	ADH1A, ADH1B, ADH1C, ADH4, ADH6, ADH7, ALDH2	10.45	18.41
KEGG:00071	Fatty acid degradation	8.27E-07	ADH1A, ADH1B, ADH1C, ADH4, ADH6, ADH7, ALDH2	15.91	28.04
KEGG:00350	Tyrosine metabolism	2.92E-06	ADH1A, ADH1B, ADH1C, ADH4, ADH6, ADH7	17.14	30.21
KEGG:00561	Glycerolipid metabolism	1.17E-02	ALDH2, DGKH, PPAP2B	5.08	8.96
KEGG:00830	Retinol metabolism	6.54E-06	ADH1A, ADH1B, ADH1C, ADH4, ADH6, ADH7, UGT2B7	10.77	18.98
KEGG:00980	Metabolism of xenobiotics by cytochrome P450	9.68E-06	ADH1A, ADH1B, ADH1C, ADH4, ADH6, ADH7, UGT2B7	9.59	16.90
KEGG:00982	Drug metabolism	7.40E-06	ADH1A, ADH1B, ADH1C, ADH4, ADH6, ADH7, UGT2B7	10.14	17.88
KEGG:04514	Cell adhesion molecules (CAMs)	8.57E-05	ALCAM, HLA-A, HLA-DPA1, HLA-G, ITGA6, NRXN1, PTPRM, VCAN	5.63	9.93
KEGG:04940	Type I diabetes mellitus	5.97E-03	HLA-A, HLA-DPA1, HLA-G	6.98	12.30
KEGG:05030	Cocaine addiction	7.63E-03	BDNF, GNAI2, GRM3	6.12	10.79
KEGG:05204	Chemical carcinogenesis	3.40E-06	ADH1A, ADH1B, ADH1C, ADH4, ADH6, ADH7, SULT1A1, UGT2B7	9.76	17.19
KEGG:05320	Autoimmune thyroid disease	8.87E-03	HLA-A, HLA-DPA1, HLA-G	5.66	9.98
KEGG:05321	Inflammatory bowel disease (IBD)	2.96E-03	HLA-DPA1, IL18R1, IL6, TGFB2	6.15	10.85
KEGG:05330	Allograft rejection	4.82E-03	HLA-A, HLA-DPA1, HLA-G	7.89	13.91
KEGG:05332	Graft-versus-host disease	8.75E-04	HLA-A, HLA-DPA1, HLA-G, IL6	9.76	17.19
KEGG:05410	Hypertrophic cardiomyopathy (HCM)	5.64E-03	CACNG6, IL6, ITGA6, TGFB2	4.82	8.49
KEGG:05416	Viral myocarditis	1.17E-02	HLA-A, HLA-DPA1, HLA-G	5.08	8.96

* P-value was adjusted by Benjamini-Hochberg methods.

### Pathway grouped network analysis

**Figure 1:**
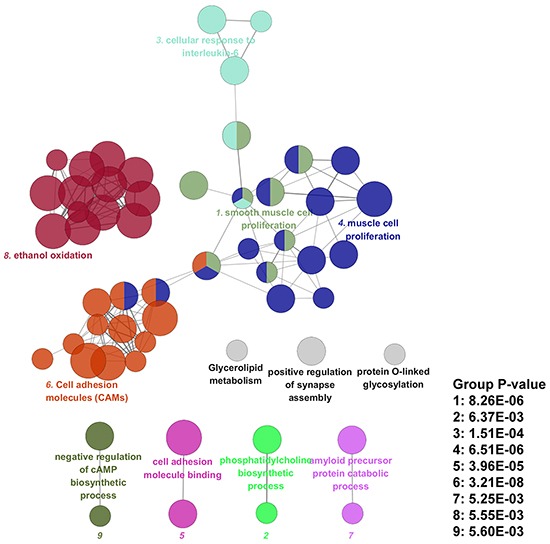
Pathway grouped network of esophageal cancer

As shown in Supplementary Table S4, a pathway grouped network was constructed with significant interacted pathways involved and 55 pathways of EC were significantly grouped into 9 sub-networks with Group P-value < 0.05. As shown in Figure 1, Group 1 included smooth muscle cell proliferation related pathways; Group 2 included phosphatidylcholine biosynthetic process related pathways; Group 3 were cellular response to interleukin-6 involved pathways; Group 4 were muscle cell proliferation related pathways; Group 5 and Group 6 was related with cell adhesion molecule binding and Cell adhesion molecules (CAMs) respectively; Group 7 was amyloid precursor protein catabolic process; Group 8 was related with ethanol oxidation and Group 9 was negative regulation of cAMP biosynthetic process. Besides, 3 significant pathways including protein O-linked glycosylation, positive regulation of synapse assembly, glycerolipid metabolism were independent and not grouped into any cluster.

## DISCUSSION

In this study, we employed an integrative genomics approach to investigate genetic risk factors and biological functions of EC. By systematic data analysis, evidence from large-scale GWAS, eQTL, pathway and network were obtained. As shown in Supplementary Figure S1, nine risk SNPs on alcohol dehydrogenase genes (eg. *ADH4*, *ADH1C*) were identified to have significantly differential gene expression levels under different genotypes on esophageal tissues including esophagus muscularis and esophagus mucosa, as alcohol drinking has been considered as an important risk factor of EC [2], and previous animal studies also demonstrated impairment of aldehyde dehydrogenase could increase accumulation of acetaldehyde-derived DNA damage in the esophagus after ethanol ingestion [7]. Our eQTL results indicated compared with non-risk alleles/genotypes, risk alleles/genotypes of these GWAS identified SNPs had differential gene expression levels, thus altered expression of risk genes might contribute to the molecular mechanisms of EC and were worthy of further investigation.

By functional annotation with genome information, 211 risk SNPs were mapped into 170 genes, which were enriched into 38 significant GO terms and 17 significant KEGG pathways by pathway enrichment analysis. Then these EC related pathways were significantly grouped into 9 sub-networks according to shared risk genes among pathways. Two pathway groups related to muscle cell proliferation were identified, with genes such as *FGFR2* and *FOXP1* involved. In accordance with our results, have shown *FGFR2* are able to promote tumor development and progression in esophageal carcinoma [8] and *FOXP1*, as a member of Forkhead-box (FOX) family genes, was reported to be associated with poor prognosis of multi-cancer [9]. In addition, the alcohol related pathway group including alcohol dehydrogenase (NAD) activity, aldehyde dehydrogenase (NAD(P)+) activity, ethanol oxidation, alcohol catabolic process was identified, which provided genetic evidence and biological explanation for the risk of alcohol drinking on development of EC [2]. Meanwhile, some interleukin-6 (IL-6) mediated immunity pathways were also grouped, such as cellular response to IL-6, T-helper cell differentiation and positive T cell selection, which also demonstrated an important involvement of *IL-6* on the development of EC [10]. Moreover, the identification of cell adhesion molecules (CAMs) related pathway groups were supported by previous studies reporting altered expression of CAMs during prognosis and tumor behavior in EC [11].

Results from pathway grouped network analysis demonstrated some pathways were shared among different groups, such as immune related pathways including T-helper cell differentiation, alpha-beta T cell activation and positive regulation of interferon-gamma production; as well as muscle development related pathways such as regulation of muscle organ development and smooth muscle cell proliferation, indicating EC related genes and pathways did not function independently, but functioned in the form of interacting with each other. Therefore, results from our study revealed the multi-gene effect on genetic basis of EC, supporting the view indicating that combined effect of multi genetic variants or genes with modest effect were also involved in genetic basis of complex disorders such as EC [5].

In conclusion, in this study, we explored genetic basis of EC by comprehensive data mining and systematical data analysis based on GWAS data, evidence from SNP, gene, gene expressions, pathway and network were identified, which might provide new insight for exploring the molecular mechanisms of EC.

## MATERIALS AND METHODS

### Identification of SNPs associated with risk of esophageal cancer

In order to identify SNPs associated with risk of esophageal cancer, GWAS of esophageal cancer were collected from GWAS catalog (https://www.genome.gov/gwastudies/), which collected all currently published GWAS of various traits. Besides, we also searched public database of Pubmed to collect recently published GWAS of esophageal cancer. Information of GWAS studies including sample size, genotyping platform, ethnic groups, reported SNPs and their P-values were collected. Due to the polygenic risk of complex disorders, individual genetic variants may only accounted for a very small amount of the genetic risk and heritability of complex disorders such as esophageal cancer [4], in order to more comprehensively capture SNPs with small effect size, we used genetic association P-value of 5.0E−05 as a criterion for identifying SNPs that are associated with risk of esophageal cancer. To detect the power of each GWAS in identifying risk SNPs, we performed power analysis by QUNTO (http://biostats.usc.edu/Quanto.html) [12]. To comprehensively investigate the power of GWAS, three levels of risk allele frequency was assumed, which were 0.1, 0.5 and 0.9 respectively. The odds ratio was assumed as 1.20, demonstrating a “weak to moderate” gene effect, and two-tailed α  was set as 0.05.

### Functional annotation and expression quantitative trait loci (eQTL) analysis

To identify genes of SNPs and candidate regulatory SNPs at disease-associated loci, we annotated genome information to SNPs including related chromosome, genome position, allele changes, mapped genes by using data from 1000 Genomes Project [13] and ENCODE (Encyclopedia of DNA Elements) projects [14]. Genes mapped by risk SNPs were considered as risk genes of esophageal cancer.

Meanwhile, some GWAS identified SNPs had regulatory functions by causing differential gene expressions with different genotypes and understanding the functional consequence of genetic variants was essential for biological interpretation on genetic etiology of disease [15]. Expression quantitative trait locus (eQTL) analysis was the most common approach used to dissect the effects of genetic variation on gene expression. As esophageal cancer occurred in esophageal tissues, the expression effect of risk SNPs in these tissues was worthy of being investigated. To detect the potential impact of risk SNPs on gene expression in esophageal tissues, we performed eQTL analysis by investigating the tissue specific expression distributions of SNPs in diverse human tissues using the Genotype Tissue Expression portal (GTEx) [16], a database that contained RNA sequencing data from 1641 samples across 43 tissues from 175 individuals. For each tissue, significance correlations between genotypes and gene expression levels were determined by linear regression on quantile normalized gene-level expression values, with permutation-adjusted P–value < 0.05 as significance. As referred in [16], The eQTL was calculated for SNPs within ±1 Mb of the transcriptional start site (TSS) of each gene. If more than one target gene was identified for one SNP by eQTL analysis, gene with the most significant P-value was chosen.

### Pathway enrichment analysis

To investigate whether risk genes of esophageal cancer identified from GWAS were enriched in functional pathways, we performed pathway enrichment analysis. Information from Kyoto encyclopedia of genes and genomes (KEGG) database [17] and Gene ontology (GO) terms [18] was used to annotate related pathways. The pathway enrichment test was based on hypergeometric test, the P-value was corrected by Benjamini-Hochberg methods and the significance was set as 0.05. To measure the magnitude of risk gene enrichment, we calculated the fold enrichment of involved risk genes in each pathway. The fold enrichment was obtained by calculating proportion of involved risk genes versus proportion of involved genes in human genome with a total of 29960 genes in each pathway according to the method applied in [19], with a suggested threshold of fold enrichment as 1.5 and above.

### Pathway grouped network analysis

To investigate whether identified pathways were biologically interconnected, we constructed a pathway grouped network of risk genes of esophageal cancer by using a Cytoscape plug-in called “ClueGO” [20]. The relationship between pathways was defined based on their shared genes and calculated by chance corrected kappa statistics. Then the created network represented the pathways as nodes which were linked based on a predefined kappa score level. In our pathway grouped network analysis, we set the kappa score level as “0.4” as ClueGo referenced. The group P-value was determined by hypergeometric test, the P-value was corrected by Benjamini-Hochberg methods and the significance was set as 0.05. The final network was visualized by Cytoscape software (Version 3.1.1).

## SUPPLEMENTARY FIGURES AND TABLES










