# Supplementary material for: Convergent evidence from systematic analysis of GWAS revealed genetic basis of esophageal cancer
Source: Oncotarget. 2016 Jun 17;7(28):44621–9. doi: 10.18632/oncotarget.10133 (PMC5190123; doi:10.18632/oncotarget.10133)
Supplement: Supplementary file 1 [file oncotarget-07-44621-s001.pdf]

Convergent evidence from systematic analysis of GWAS revealed genetic basis of esophageal cancer

SUPPLEMENTARY FIGURE AND TABLES

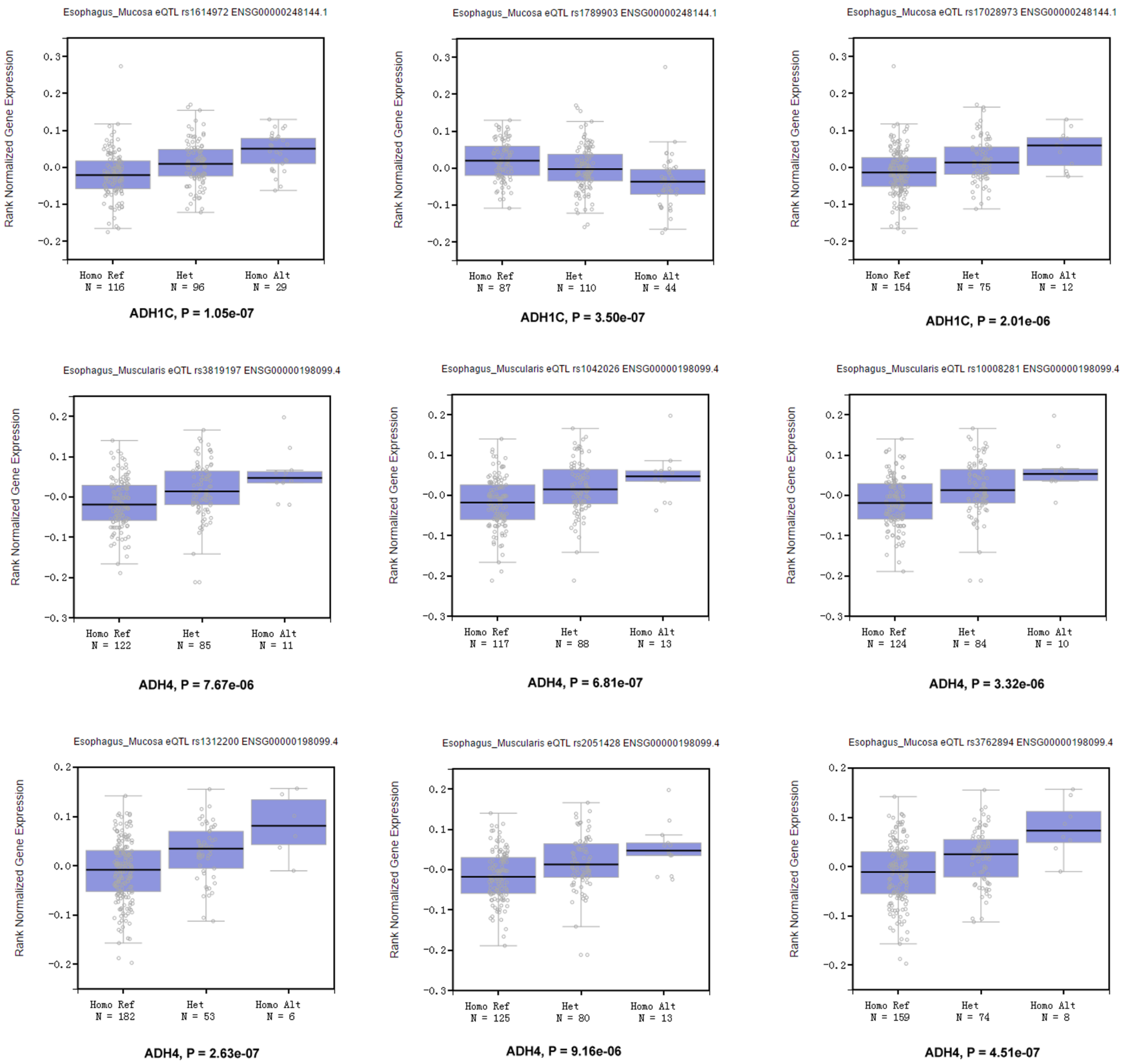

Supplementary Figure S1: Box plots of risk SNPs in gene *ADH4* and *ADH1C* by eQTL analysis. Homo Ref: Homologous referenced genotype; Het: heterologous genotype; Homo Alt: Homologous alternative genotype.

Supplementary Table S1: SNPs with GWAS P-value <5.0E-05.

See Supplementary File 1

Supplementary Table S2: Annotated information of risk SNPs of esophageal cancer.

See Supplementary File 2

Supplementary Table S3: Risk SNPs with significant gene expression changes on esophageal tissues.

See Supplementary File 3

Supplementary Table S4: Details of pathway grouped network.

See Supplementary File 4

Supplementary Table S5: Power analysis of GWAS of esophageal cancer

Study	Initial sample size	Replicated Sample size	Power of initial anlysis			Power of joint analysis (initial sample + replicated sample)		
			Assumed risk allele frequency					
			0.1	0.5	0.9	0.1	0.5	0.9
Levine DM[21]	4725	7785	99%	99%	99%	99%	99%	99%
Jin G[22]	6037	14442	99%	99%	99%	99%	99%	99%
Wu C[23]	4075	16712	99%	99%	99%	99%	99%	99%
Wu C[24]	4075	8143	99%	99%	99%	99%	99%	99%
Abnet CC[25]	3998	NA	96%	99%	93%	/	/	/
Wang LD[26]	2810	19526	92%	99%	87%	99%	99%	99%
Cui R[27]	1109	2680	69%	97%	61%	99%	99%	99%
