# Supplementary material for: Convergent evidence from systematic analysis of GWAS revealed genetic basis of esophageal cancer
Source: Oncotarget. 2016 Jun 17;7(28):44621–9. doi: 10.18632/oncotarget.10133 (PMC5190123; doi:10.18632/oncotarget.10133)
Supplement: Supplementary file 2 [file oncotarget-07-44621-s002.docx]

**Supplementary data**

**Table S1** SNPs with GWAS P-value <5.0E-05

| **Study** | **Reference** | **Reported SNPs** | **GWAS P-value** | **Odds ratio** |
| --- | --- | --- | --- | --- |
| Levine DM | [21] | rs10419226 | 8.00E-07 | 1.19 |
|  |  | rs10423674 | 1.46E-06 | 0.83 |
|  |  | rs11789015 | 2.00E-07 | 1.23 |
|  |  | rs1547073 | 9.99E-06 | 1.22 |
|  |  | rs17172185 | 5.00E-06 | 1.47 |
|  |  | rs2178146 | 4.00E-06 | 1.18 |
|  |  | rs2342002 | 9.00E-07 | 1.22 |
|  |  | rs2687201 | 6.00E-07 | 1.20 |
|  |  | rs4610302 | 4.00E-06 | 1.18 |
|  |  | rs4800353 | 1.13E-05 | 0.79 |
|  |  | rs4880498 | 2.67E-05 | 0.86 |
|  |  | rs6479527 | 2.00E-06 | 1.18 |
|  |  | rs9837992 | 3.63E-05 | 1.17 |
| Jin G | [22] | rs2285947 | 3.00E-06 | 1.14 |
| Wu C | [23] | rs1000668 | 1.09E-07 | 1.27 |
|  |  | rs10131468 | 7.24E-06 | 1.29 |
|  |  | rs1017913 | 4.78E-05 | 1.46 |
|  |  | rs10191793 | 6.05E-07 | 0.80 |
|  |  | rs10274928 | 7.70E-07 | 2.34 |
|  |  | rs1029686 | 4.48E-05 | 0.80 |
|  |  | rs1042026 | 3.00E-07 | 1.27 |
|  |  | rs10501087 | 1.69E-05 | 0.82 |
|  |  | rs10502639 | 2.20E-06 | 0.70 |
|  |  | rs10502995 | 6.88E-07 | 0.80 |
|  |  | rs10517215 | 1.88E-05 | 0.80 |
|  |  | rs10788679 | 5.11E-06 | 1.40 |
|  |  | rs10863469 | 3.75E-05 | 1.21 |
|  |  | rs10913801 | 2.22E-06 | 0.75 |
|  |  | rs10920724 | 3.11E-05 | 0.79 |
|  |  | rs11051196 | 5.25E-07 | 0.80 |
|  |  | rs1114737 | 3.47E-05 | 1.21 |
|  |  | rs11169561 | 1.40E-06 | 0.80 |
|  |  | rs11254327 | 7.02E-07 | 1.26 |
|  |  | rs11562986 | 4.93E-06 | 0.77 |
|  |  | rs11776675 | 1.82E-07 | 1.43 |
|  |  | rs11843987 | 2.88E-07 | 0.71 |
|  |  | rs11929651 | 4.49E-05 | 0.75 |
|  |  | rs11934740 | 3.74E-05 | 0.81 |
|  |  | rs11972901 | 9.23E-07 | 1.26 |
|  |  | rs1229977 | 3.22E-06 | 1.37 |
|  |  | rs12413624 | 2.61E-07 | 1.27 |
|  |  | rs12438490 | 1.02E-07 | 1.28 |
|  |  | rs12443881 | 2.45E-06 | 1.36 |
|  |  | rs12502693 | 1.56E-05 | 1.23 |
|  |  | rs12560500 | 4.12E-06 | 0.80 |
|  |  | rs1264616 | 5.45E-06 | 0.81 |
|  |  | rs12648830 | 2.35E-05 | 1.21 |
|  |  | rs12674026 | 3.18E-06 | 1.46 |
|  |  | rs1323135 | 2.02E-07 | 1.32 |
|  |  | rs1429139 | 2.54E-06 | 0.81 |
|  |  | rs1430055 | 2.79E-07 | 1.32 |
|  |  | rs1437787 | 2.67E-06 | 1.45 |
|  |  | rs1453029 | 2.42E-07 | 0.75 |
|  |  | rs1510989 | 1.85E-05 | 0.82 |
|  |  | rs151181 | 2.48E-07 | 1.39 |
|  |  | rs1548418 | 3.87E-07 | 1.27 |
|  |  | rs1570477 | 2.32E-07 | 1.38 |
|  |  | rs1614972 | 1.34E-06 | 1.27 |
|  |  | rs1655900 | 2.27E-07 | 1.86 |
|  |  | rs16834075 | 2.55E-05 | 0.76 |
|  |  | rs16901549 | 6.42E-07 | 0.62 |
|  |  | rs16991006 | 4.24E-05 | 1.40 |
|  |  | rs17002407 | 2.91E-05 | 0.82 |
|  |  | rs17028973 | 4.61E-07 | 1.26 |
|  |  | rs17033 | 2.80E-07 | 1.40 |
|  |  | rs17034964 | 3.64E-05 | 1.21 |
|  |  | rs17114119 | 2.78E-05 | 0.73 |
|  |  | rs17147265 | 5.93E-06 | 0.68 |
|  |  | rs17156432 | 1.90E-05 | 0.82 |
|  |  | rs17205706 | 4.71E-05 | 1.47 |
|  |  | rs1737076 | 9.85E-07 | 1.26 |
|  |  | rs17450420 | 5.00E-08 | 1.70 |
|  |  | rs174547 | 1.80E-05 | 0.82 |
|  |  | rs17578989 | 2.23E-05 | 1.24 |
|  |  | rs17637472 | 1.06E-06 | 0.75 |
|  |  | rs17728461 | 2.19E-05 | 1.26 |
|  |  | rs17761864 | 2.00E-11 | 1.21 |
|  |  | rs17781266 | 2.99E-05 | 0.82 |
|  |  | rs1789903 | 3.35E-06 | 1.41 |
|  |  | rs1872645 | 4.07E-06 | 0.72 |
|  |  | rs1893883 | 1.23E-05 | 1.31 |
|  |  | rs1968752 | 1.20E-06 | 1.44 |
|  |  | rs1976053 | 9.21E-06 | 1.23 |
|  |  | rs2239612 | 6.00E-14 | 1.21 |
|  |  | rs2239815 | 4.00E-15 | 1.18 |
|  |  | rs2252257 | 1.68E-06 | 1.25 |
|  |  | rs2252641 | 5.63E-07 | 1.31 |
|  |  | rs2282751 | 7.20E-06 | 1.23 |
|  |  | rs2286276 | 8.01E-07 | 1.40 |
|  |  | rs2347540 | 2.50E-05 | 1.22 |
|  |  | rs2373298 | 2.26E-06 | 0.80 |
|  |  | rs2378002 | 1.99E-07 | 0.78 |
|  |  | rs2409764 | 5.78E-07 | 1.57 |
|  |  | rs26704 | 9.38E-07 | 1.28 |
|  |  | rs2803270 | 9.16E-07 | 1.26 |
|  |  | rs2803533 | 1.51E-06 | 0.78 |
|  |  | rs2845573 | 3.99E-05 | 0.81 |
|  |  | rs2847281 | 2.00E-11 | 1.20 |
|  |  | rs2856816 | 2.32E-06 | 0.81 |
|  |  | rs2984526 | 2.16E-07 | 1.26 |
|  |  | rs3077 | 6.66E-06 | 1.23 |
|  |  | rs3096337 | 3.30E-05 | 1.23 |
|  |  | rs310518 | 3.78E-05 | 0.79 |
|  |  | rs3398 | 5.28E-07 | 1.26 |
|  |  | rs357271 | 6.94E-06 | 0.68 |
|  |  | rs3805322 | 6.00E-07 | 1.23 |
|  |  | rs3807390 | 7.82E-07 | 1.66 |
|  |  | rs3825402 | 1.17E-06 | 0.80 |
|  |  | rs41269947 | 3.58E-06 | 0.79 |
|  |  | rs4129666 | 1.40E-05 | 0.73 |
|  |  | rs41475647 | 5.82E-06 | 0.82 |
|  |  | rs4148641 | 8.98E-07 | 1.46 |
|  |  | rs4283388 | 1.18E-07 | 1.58 |
|  |  | rs4348786 | 1.57E-05 | 1.23 |
|  |  | rs4503732 | 4.56E-07 | 0.79 |
|  |  | rs457864 | 2.78E-06 | 0.81 |
|  |  | rs475010 | 2.28E-05 | 0.78 |
|  |  | rs4785204 | 2.00E-20 | 1.24 |
|  |  | rs4788074 | 5.44E-07 | 1.42 |
|  |  | rs4822983 | 2.00E-22 | 1.27 |
|  |  | rs4851005 | 7.61E-07 | 0.79 |
|  |  | rs4872018 | 1.57E-07 | 0.77 |
|  |  | rs4970821 | 3.06E-07 | 1.26 |
|  |  | rs5020127 | 2.85E-06 | 1.24 |
|  |  | rs5743592 | 4.58E-05 | 0.83 |
|  |  | rs5762788 | 1.27E-05 | 1.22 |
|  |  | rs581037 | 4.66E-07 | 1.51 |
|  |  | rs5996074 | 2.70E-07 | 0.79 |
|  |  | rs6134846 | 3.46E-05 | 1.23 |
|  |  | rs6421018 | 3.18E-05 | 1.23 |
|  |  | rs6503659 | 3.00E-16 | 1.27 |
|  |  | rs6565300 | 2.85E-06 | 1.41 |
|  |  | rs6585737 | 3.74E-06 | 1.79 |
|  |  | rs6817108 | 2.40E-05 | 1.25 |
|  |  | rs6884677 | 5.73E-06 | 1.39 |
|  |  | rs6894687 | 2.07E-07 | 0.72 |
|  |  | rs701464 | 5.87E-06 | 1.32 |
|  |  | rs7022441 | 1.40E-07 | 1.27 |
|  |  | rs7206735 | 3.49E-07 | 1.29 |
|  |  | rs7334071 | 3.46E-06 | 1.33 |
|  |  | rs737909 | 2.31E-05 | 1.34 |
|  |  | rs737980 | 3.24E-06 | 0.78 |
|  |  | rs7505928 | 1.20E-06 | 0.71 |
|  |  | rs753544 | 3.15E-07 | 1.36 |
|  |  | rs7626795 | 3.63E-06 | 1.31 |
|  |  | rs7642279 | 3.87E-06 | 1.54 |
|  |  | rs7679999 | 3.23E-05 | 1.23 |
|  |  | rs7699182 | 3.89E-05 | 0.80 |
|  |  | rs777241 | 1.41E-06 | 0.67 |
|  |  | rs7801967 | 1.79E-06 | 1.25 |
|  |  | rs7851133 | 1.24E-07 | 0.79 |
|  |  | rs8057779 | 2.67E-07 | 1.31 |
|  |  | rs896570 | 3.88E-07 | 0.79 |
|  |  | rs902907 | 4.08E-07 | 1.26 |
|  |  | rs907091 | 3.38E-07 | 1.28 |
|  |  | rs9288520 | 4.00E-11 | 1.33 |
|  |  | rs9408157 | 2.53E-06 | 1.24 |
|  |  | rs9517529 | 1.74E-06 | 0.80 |
|  |  | rs9547964 | 1.59E-06 | 1.62 |
|  |  | rs9547991 | 8.09E-07 | 1.63 |
|  |  | rs9566910 | 2.81E-07 | 0.79 |
|  |  | rs9651118 | 2.48E-05 | 0.81 |
|  |  | rs9852888 | 2.48E-05 | 0.79 |
|  |  | rs9864042 | 3.19E-06 | 0.77 |
|  |  | rs9986 | 5.81E-06 | 1.26 |
| Wu C | [24] | rs11066280 | 2.00E-15 | 1.30 |
|  |  | rs2074356 | 2.00E-31 | 1.56 |
|  |  | rs10058728 | 5.00E-09 | 2.04 |
|  |  | rs6772209 | 3.00E-07 | 1.49 |
|  |  | rs9868873 | 1.00E-07 | 1.19 |
|  |  | rs8030672 | 1.00E-11 | 1.56 |
|  |  | rs2274223 | 4.00E-20 | 1.34 |
|  |  | rs10052657 | 2.00E-19 | 1.49 |
|  |  | rs11066015 | 7.00E-21 | 1.38 |
|  |  | rs2014300 | 8.00E-22 | 1.43 |
|  |  | rs10484761 | 7.00E-12 | 1.33 |
| Abnet CC | [25] | rs11187842 | 1.66E-06 | 1.37 |
|  |  | rs1547014 | 2.79E-07 | 1.33 |
|  |  | rs2274223 | 7.75E-07 | 1.33 |
|  |  | rs3765524 | 2.00E-09 | 1.35 |
|  |  | rs3781264 | 7.14E-07 | 1.38 |
|  |  | rs738722 | 1.00E-08 | 1.30 |
|  |  | rs753724 | 1.35E-06 | 1.37 |
| Wang LD | [26] | rs4908343 | 1.21E-07 | 0.73 |
|  |  | rs11134032 | 1.25E-06 | 1.31 |
|  |  | rs6860112 | 1.03E-06 | 1.31 |
|  |  | rs1346291 | 3.70E-07 | 0.72 |
|  |  | rs12263737 | 2.07E-09 | 1.47 |
|  |  | rs2274223 | 2.81E-09 | 1.47 |
|  |  | rs12580487 | 8.67E-06 | 1.31 |
|  |  | rs7230870 | 2.41E-04 | 1.23 |
|  |  | rs4891058 | 5.53E-05 | 1.26 |
|  |  | rs8102476 | 1.56E-06 | 0.75 |
|  |  | rs462094 | 1.20E-05 | 1.34 |
|  |  | rs6140125 | 2.36E-12 | 0.65 |
|  |  | rs13042395 | 1.51E-11 | 0.66 |
|  |  | rs6098279 | 1.81E-06 | 0.61 |
|  |  | rs6023640 | 1.34E-07 | 0.56 |
|  |  | rs2830856 | 2.71E-06 | 1.30 |
|  |  | rs11090042 | 1.78E-06 | 1.32 |
|  |  | rs5753220 | 7.85E-10 | 0.71 |
| Cui R | [27] | rs671 | 3.00E-24 | 1.67 |
|  |  | rs1229984 | 8.00E-24 | 1.79 |
|  |  | rs3782886 | 5.46E-16 | 1.67 |
|  |  | rs3819197 | 3.32E-14 | 1.85 |
|  |  | rs2051428 | 8.84E-14 | 1.80 |
|  |  | rs1159918 | 1.08E-13 | 1.88 |
|  |  | rs1042026 | 1.72E-13 | 1.83 |
|  |  | rs10008281 | 4.19E-13 | 1.79 |
|  |  | rs1442490 | 1.07E-09 | 1.54 |
|  |  | rs1312200 | 5.35E-08 | 1.46 |
|  |  | rs994771 | 6.15E-08 | 1.50 |
|  |  | rs3762894 | 1.38E-07 | 1.45 |
