# Supplementary material for: Convergent evidence from systematic analysis of GWAS revealed genetic basis of esophageal cancer
Source: Oncotarget. 2016 Jun 17;7(28):44621–9. doi: 10.18632/oncotarget.10133 (PMC5190123; doi:10.18632/oncotarget.10133)
Supplement: Supplementary file 3 [file oncotarget-07-44621-s003.docx]

**Table S2** Annotated information of risk SNPs of esophageal cancer

| **rsID** | **chromosome** | **pos_hg38** | **reference allele** | **altered allele** | **Annotated gene** |
| --- | --- | --- | --- | --- | --- |
| rs10419226 | 19 | 18692362 | T | G | CRTC1 |
| rs10423674 | 19 | 18707093 | C | A | CRTC1 |
| rs11789015 | 9 | 93953746 | A | G | BARX1 |
| rs1547073 | 6 | 61835688 | G | A | KHDRBS2 |
| rs17172185 | 7 | 43247240 | T | C | HECW1 |
| rs2178146 | 16 | 86430089 | T | C | LOC400550 |
| rs2342002 | 6 | 61285855 | G | A | MTRNR2L9 |
| rs2687201 | 3 | 70879779 | A | C | FOXP1 |
| rs4610302 | 4 | 87478958 | A | G | SPARCL1 |
| rs4800353 | 18 | 22074176 | A | G | GATA6 |
| rs4880498 | 10 | 1360689 | T | G | ADARB2 |
| rs6479527 | 9 | 94096129 | G | A | PTPDC1 |
| rs9837992 | 3 | 70910287 | G | A | FOXP1 |
| rs2285947 | 7 | 21544470 | G | A | DNAH11 |
| rs1000668 | 9 | 72790943 | T | A | TMC1 |
| rs10131468 | 14 | 82599694 | T | C | SEL1L |
| rs1017913 | 3 | 105519771 | G | T | ALCAM |
| rs10191793 | 2 | 106809420 | A | G | ST6GAL2 |
| rs10274928 | 7 | 28102469 | A | G | JAZF1 |
| rs1029686 | 1 | 230492133 | T | C | PGBD5 |
| rs1042026 | 4 | 99307309 | T | C | ADH1B |
| rs10501087 | 11 | 27648561 | T | C | BDNF-AS1 |
| rs10502639 | 18 | 34932686 | C | T | DTNA |
| rs10502995 | 18 | 55138645 | G | A | TCF4 |
| rs10517215 | 4 | 30772461 | C | A | PCDH7 |
| rs10788679 | 1 | 17590467 | A | G | ARHGEF10L |
| rs10863469 | 1 | 219604126 | T | C | RNU5F |
| rs10913801 | 1 | 179502615 | T | G | AXDND1 |
| rs10920724 | 1 | 190468058 | G | A | FAM5C |
| rs11051196 | 12 | 30980130 | C | T | TSPAN11 |
| rs1114737 | 1 | 55499740 | T | G | MIR4422 |
| rs11169561 | 12 | 50792167 | C | T | ATF1 |
| rs11254327 | 10 | 17006709 | C | T | CUBN |
| rs11562986 | 2 | 51838829 | G | A | NRXN1 |
| rs11776675 | 8 | 94163787 | T | C | CDH17 |
| rs11843987 | 13 | 113589954 | A | G | TFDP1 |
| rs11929651 | 3 | 29447544 | G | A | RBMS3 |
| rs11934740 | 4 | 128961593 | C | T | SCLT1 |
| rs11972901 | 7 | 85966605 | A | G | GRM3 |
| rs1229977 | 4 | 99281257 | T | C | ADH1A |
| rs12413624 | 10 | 118519432 | T | A | PRLHR |
| rs12438490 | 15 | 28015163 | C | T | OCA2 |
| rs12443881 | 16 | 28830456 | C | T | ATXN2L |
| rs12502693 | 4 | 75268194 | T | C | LOC441025 |
| rs1264616 | 6 | 30296530 | C | T | HCG18 |
| rs12648830 | 4 | 69075436 | C | A | UGT2B7 |
| rs12674026 | 7 | 29677069 | G | A | LOC646762 |
| rs1323135 | 20 | 1377208 | G | T | FKBP1A |
| rs1429139 | 4 | 147362952 | T | A | EDNRA |
| rs1430055 | 2 | 16499521 | G | A | FAM49A |
| rs1437787 | 2 | 215453514 | T | C | FN1 |
| rs1453029 | 18 | 41000207 | T | C | KC6 |
| rs1510989 | 16 | 49261696 | T | C | CBLN1 |
| rs151181 | 16 | 28479196 | T | C | CLN3 |
| rs1548418 | 7 | 22685795 | C | A | IL6 |
| rs1570477 | 9 | 657340 | G | A | KANK1 |
| rs1614972 | 4 | 99336998 | C | T | ADH1C |
| rs1655900 | 6 | 29948841 | G | A | HLA-A |
| rs16834075 | 2 | 153705112 | C | T | GALNT13 |
| rs16901549 | 8 | 126583939 | G | T | FAM84B |
| rs16991006 | 4 | 35374407 | T | C | ARAP2 |
| rs17002407 | 22 | 41348330 | A | G | ZC3H7B |
| rs17028973 | 4 | 99401629 | T | C | ADH7 |
| rs17033 | 4 | 99307788 | T | C | ADH1B |
| rs17034964 | 4 | 156610335 | A | G | PDGFC |
| rs17114119 | 1 | 56571287 | C | T | PPAP2B |
| rs17147265 | 7 | 22765320 | A | C | IL6 |
| rs17156432 | 7 | 28122786 | T | C | JAZF1 |
| rs17205706 | 5 | 83100950 | A | G | XRCC4 |
| rs1737076 | 6 | 29757544 | A | G | IFITM4P |
| rs17450420 | 13 | 104386796 | A | G | DAOA-AS1 |
| rs174547 | 11 | 61803311 | T | C | FADS1 |
| rs17578989 | 1 | 234602243 | T | C | IRF2BP2 |
| rs17637472 | 17 | 49384071 | G | A | PHB |
| rs17728461 | 22 | 30202563 | C | G | HORMAD2 |
| rs17761864 | 17 | 2268343 | C | A | SMG6 |
| rs17781266 | 9 | 24075783 | C | G | ELAVL2 |
| rs1789903 | 4 | 99340884 | C | G | ADH1C |
| rs1872645 | 12 | 24814278 | C | T | BCAT1 |
| rs1893883 | 4 | 99203559 | C | G | ADH6 |
| rs1968752 | 16 | 28620264 | T | G | SULT1A1 |
| rs1976053 | 17 | 67836886 | C | G | BPTF |
| rs2239612 | 3 | 187075454 | G | A | ST6GAL1 |
| rs2239815 | 22 | 28796682 | T | C | XBP1 |
| rs2252257 | 22 | 18157533 | G | A | USP18 |
| rs2252641 | 2 | 145043894 | T | C | DKFZp686O1327 |
| rs2282751 | 3 | 50254353 | G | A | GNAI2 |
| rs2286276 | 7 | 73573024 | C | T | TBL2 |
| rs2347540 | 1 | 75653783 | G | A | SLC44A5 |
| rs2373298 | 2 | 37760049 | G | T | CDC42EP3 |
| rs2378002 | 1 | 218749016 | G | T | TGFB2 |
| rs2409764 | 8 | 11423764 | A | G | FAM167A |
| rs26704 | 5 | 59237827 | A | G | PDE4D |
| rs2803270 | 1 | 48565566 | C | T | AGBL4 |
| rs2803533 | 1 | 147482924 | G | A | LOC100289211 |
| rs2845573 | 11 | 61834436 | A | G | FADS2 |
| rs2847281 | 18 | 12821594 | A | G | PTPN2 |
| rs2856816 | 6 | 33077723 | T | C | HLA-DPA1 |
| rs2984526 | 9 | 72372344 | C | G | ZFAND5 |
| rs3077 | 6 | 33065245 | A | G | HLA-DPA1 |
| rs3096337 | 3 | 195806461 | C | T | MUC4 |
| rs310518 | 5 | 83523300 | G | A | VCAN |
| rs3398 | 9 | 72354160 | G | A | ZFAND5 |
| rs357271 | 5 | 38876471 | T | C | OSMR |
| rs3805322 | 4 | 99135847 | A | G | ADH4 |
| rs3807390 | 7 | 38533831 | T | C | AMPH |
| rs3825402 | 12 | 50733955 | T | C | DIP2B |
| rs4129666 | 8 | 127591164 | T | C | LOC727677 |
| rs41475647 | 9 | 111476454 | C | T | KIAA0368 |
| rs4148641 | 11 | 17399926 | G | A | ABCC8 |
| rs4283388 | 2 | 172374994 | C | T | ITGA6 |
| rs4348786 | 1 | 216908941 | C | T | ESRRG |
| rs4503732 | 15 | 46350065 | C | A | SQRDL |
| rs457864 | 21 | 26799607 | A | C | ADAMTS1 |
| rs475010 | 4 | 134737528 | T | C | PABPC4L |
| rs4785204 | 16 | 50069823 | C | T | HEATR3 |
| rs4788074 | 16 | 28582276 | G | A | CCDC101 |
| rs4822983 | 22 | 28719078 | C | T | CHEK2 |
| rs4851005 | 2 | 102395092 | C | T | IL18R1 |
| rs4872018 | 8 | 22794657 | G | T | PEBP4 |
| rs4970821 | 1 | 108316692 | T | C | NBPF4 |
| rs5020127 | 11 | 27440232 | T | C | LGR4 |
| rs5743592 | 4 | 38801442 | A | G | TLR1 |
| rs5762788 | 22 | 28778782 | C | A | CCDC117 |
| rs581037 | 7 | 17050319 | C | T | AGR3 |
| rs5996074 | 22 | 41840333 | G | A | SREBF2 |
| rs6134846 | 20 | 1403321 | A | G | FKBP1A |
| rs6421018 | 8 | 139200716 | T | C | COL22A1 |
| rs6503659 | 17 | 41741012 | A | T | HAP1 |
| rs6565300 | 16 | 28981728 | A | C | SPNS1 |
| rs6585737 | 10 | 121455759 | C | T | FGFR2 |
| rs6817108 | 4 | 8692663 | T | C | CPZ |
| rs6884677 | 5 | 170023007 | C | G | DOCK2 |
| rs6894687 | 5 | 113407256 | G | C | MCC |
| rs701464 | 15 | 46578657 | C | T | SEMA6D |
| rs7022441 | 9 | 72521873 | G | A | TMC1 |
| rs7206735 | 16 | 50114597 | T | C | HEATR3 |
| rs7334071 | 13 | 99878957 | C | T | CLYBL |
| rs737909 | 22 | 29992769 | T | G | MTMR3 |
| rs737980 | 22 | 29541250 | C | A | THOC5 |
| rs7505928 | 18 | 8271137 | C | A | PTPRM |
| rs753544 | 6 | 29812752 | C | T | HLA-G |
| rs7626795 | 3 | 190632672 | A | G | IL1RAP |
| rs7642279 | 3 | 147737622 | A | G | ZIC1 |
| rs7679999 | 4 | 181849709 | T | C | MGC45800 |
| rs7699182 | 4 | 130370493 | A | G | C4orf33 |
| rs777241 | 3 | 126611067 | A | G | TXNRD3 |
| rs7801967 | 7 | 28205676 | C | T | LOC100128081 |
| rs7851133 | 9 | 21846328 | C | T | MTAP |
| rs8057779 | 16 | 78349243 | C | G | WWOX |
| rs896570 | 10 | 47145727 | G | A | PTPN20B |
| rs902907 | 9 | 79199649 | G | A | TLE4 |
| rs907091 | 17 | 39765489 | C | T | IKZF3 |
| rs9288520 | 2 | 216616548 | G | A | IGFBP2 |
| rs9408157 | 9 | 29919133 | A | G | MIR873 |
| rs9517529 | 13 | 98989723 | C | T | DOCK9 |
| rs9547964 | 13 | 37577065 | T | G | POSTN |
| rs9547991 | 13 | 37643278 | T | C | TRPC4 |
| rs9566910 | 13 | 42076866 | G | A | DGKH |
| rs9651118 | 1 | 11802157 | T | C | MTHFR |
| rs9852888 | 3 | 55769407 | A | T | ERC2 |
| rs9864042 | 3 | 85774411 | G | A | CADM2 |
| rs9986 | 3 | 58294679 | A | G | ABHD6 |
| rs11066280 | 12 | 112379979 | T | A | C12orf51 |
| rs2074356 | 12 | 112207597 | G | A | C12orf51 |
| rs10058728 | 5 | 149524529 | A | T | CSNK1A1 |
| rs6772209 | 3 | 175929377 | G | A | NAALADL2 |
| rs9868873 | 3 | 123012063 | G | A | SEMA5B |
| rs8030672 | 15 | 68766745 | T | A | ANP32A |
| rs2274223 | 10 | 94306584 | A | G | PLCE1 |
| rs10052657 | 5 | 59111944 | C | A | PDE4D |
| rs11066015 | 12 | 111730205 | G | A | ACAD10 |
| rs2014300 | 21 | 34985564 | A | G | RUNX1 |
| rs10484761 | 6 | 40834522 | T | C | UNC5CL |
| rs11187842 | 10 | 94292754 | C | T | PLCE1 |
| rs1547014 | 22 | 28704723 | T | C | CHEK2 |
| rs2274223 | 10 | 94306584 | A | G | PLCE1 |
| rs3765524 | 10 | 94298541 | C | T | PLCE1 |
| rs3781264 | 10 | 94310618 | A | G | PLCE1 |
| rs738722 | 22 | 28734024 | T | C | CHEK2 |
| rs753724 | 10 | 94291660 | G | T | PLCE1 |
| rs4908343 | 1 | 27605187 | G | A | AHDC1 |
| rs11134032 | 5 | 4117455 | T | C | IRX1 |
| rs6860112 | 5 | 4136041 | T | C | IRX1 |
| rs1346291 | 8 | 81339729 | A | G | FABP5 |
| rs12263737 | 10 | 94285156 | G | A | LOC100128054 |
| rs2274223 | 10 | 94306584 | A | G | PLCE1 |
| rs12580487 | 12 | 130220405 | C | T | FZD10 |
| rs7230870 | 18 | 75317453 | A | G | TSHZ1 |
| rs4891058 | 18 | 75324842 | C | T | TSHZ1 |
| rs8102476 | 19 | 38244973 | C | T | PPP1R14A |
| rs462094 | 19 | 54014014 | A | G | CACNG6 |
| rs6140125 | 20 | 764903 | C | A | C20orf54 |
| rs13042395 | 20 | 773867 | C | T | C20orf54 |
| rs6098279 | 20 | 54828759 | T | C | DOK5 |
| rs6023640 | 20 | 54842266 | T | G | DOK5 |
| rs2830856 | 21 | 27340495 | T | C | ADAMTS5 |
| rs11090042 | 22 | 22347473 | G | A | LOC96610 |
| rs5753220 | 22 | 30590363 | T | C | PES1 |
| rs671 | 12 | 111803962 | G | A | ALDH2 |
| rs1229984 | 4 | 99318162 | T | C | ADH1B |
| rs3782886 | 12 | 111672685 | T | C | BRAP |
| rs3819197 | 4 | 99279352 | C | T | ADH1A |
| rs2051428 | 4 | 99202029 | A | G | LOC100507053 |
| rs1159918 | 4 | 99321852 | A | C | ADH1B |
| rs1042026 | 4 | 99307309 | T | C | ADH1B |
| rs10008281 | 4 | 99221145 | C | A,G,T | LOC100507053 |
| rs1442490 | 4 | 99405246 | G | A | ADH7 |
| rs1312200 | 4 | 99091206 | A | G | LOC100507053 |
| rs994771 | 4 | 99406646 | G | A | ADH7 |
| rs3762894 | 4 | 99144933 | T | C | LOC100507053 |
