# Supplementary material for: Convergent evidence from systematic analysis of GWAS revealed genetic basis of esophageal cancer
Source: Oncotarget. 2016 Jun 17;7(28):44621–9. doi: 10.18632/oncotarget.10133 (PMC5190123; doi:10.18632/oncotarget.10133)
Supplement: Supplementary file 4 [file oncotarget-07-44621-s004.docx]

**Table S3** Risk SNPs with significant gene expression changes on esophageal tissues

| **rsID** | **Results of eQTL analysis (Tissue, gene, P-value)** |
| --- | --- |
| rs4148641 | Esophagus_Muscularis,KCNJ11,1.37227608978201e-06 |
| rs3819197 | Esophagus_Muscularis,ADH4,7.67049950181993e-06 |
| rs1042026 | Esophagus_Muscularis,ADH4,6.808647273704e-07 |
| rs1614972 | Esophagus_Mucosa,ADH1C,1.0526232229263e-07 |
| rs1789903 | Esophagus_Mucosa,ADH1C,3.50001671456339e-07 |
| rs17028973 | Esophagus_Mucosa,ADH1C,2.00559695022001e-06 |
| rs4788074 | Esophagus_Mucosa,SULT1A1,1.86071102131987e-15 |
| rs151181 | Esophagus_Mucosa,SULT1A1,2.77879360790264e-12 |
| rs2285947 | Esophagus_Mucosa,DNAH11,9.36179192461322e-25 |
| rs174547 | Esophagus_Mucosa,FADS1,4.57146641951091e-10 |
| rs2282751 | Esophagus_Mucosa,CYB561D2,1.83713075296317e-06 |
| rs1264616 | Esophagus_Mucosa,HCG17,7.54523270254094e-08 |
| rs4785204 | Esophagus_Muscularis,HEATR3,6.86406771788705e-09 |
| rs7206735 | Esophagus_Gastroesophageal_Junction,HEATR3,1.70602963811747e-09 |
| rs1655900 | Esophagus_Gastroesophageal_Junction,ZFP57,5.36529126016567e-08;Esophagus_Mucosa,ZFP57,6.02867782815496e-20;Esophagus_Muscularis,ZFP57,1.75074342923972e-15 |
| rs2856816 | Esophagus_Muscularis,HLA-DPB1,2.16059524325523e-09 |
| rs3077 | Esophagus_Gastroesophageal_Junction,HLA-DPA1,1.02062253142422e-13;Esophagus_Muscularis,HLA-DPA1,1.24425979735469e-36 |
| rs753544 | Esophagus_Gastroesophageal_Junction,IFITM4P,1.45845830679837e-06;Esophagus_Muscularis,IFITM4P,4.22080497381163e-09 |
| rs1737076 | Esophagus_Gastroesophageal_Junction,IFITM4P,1.30496867293068e-09;Esophagus_Muscularis,IFITM4P,1.61143878294532e-11 |
| rs907091 | Esophagus_Mucosa,GSDMB,5.18713098977917e-07 |
| rs5020127 | Esophagus_Mucosa,LIN7C,3.75342463118428e-11 |
| rs12263737 | Esophagus_Muscularis,NOC3L,1.14419481983103e-05 |
| rs10008281 | Esophagus_Muscularis,ADH4,3.32116736745308e-06 |
| rs1312200 | Esophagus_Mucosa,ADH4,2.62744829977313e-07 |
| rs2051428 | Esophagus_Muscularis,ADH4,9.16102862303983e-06 |
| rs3762894 | Esophagus_Mucosa,ADH4,4.51370172594727e-07 |
| rs3096337 | Esophagus_Muscularis,MUC20,5.55465556585192e-06 |
| rs5753220 | Esophagus_Muscularis,TCN2,1.85359938926781e-06 |
| rs2274223 | Esophagus_Muscularis,NOC3L,6.83658720250696e-06 |
| rs3765524 | Esophagus_Muscularis,NOC3L,3.46421152104094e-06 |
| rs3781264 | Esophagus_Muscularis,NOC3L,6.91988920694109e-09 |
| rs8102476 | Esophagus_Gastroesophageal_Junction,CATSPERG,7.22592195604703e-07 |
| rs11934740 | Esophagus_Mucosa,C4orf33,9.88566516322123e-07 |
| rs2347540 | Esophagus_Muscularis,MSH4,8.17024298427722e-07 |
| rs17761864 | Esophagus_Gastroesophageal_Junction,SRR,5.67252480911609e-15 |
| rs6565300 | Esophagus_Mucosa,SULT1A1,4.12851449866422e-07 |
| rs5996074 | Esophagus_Mucosa,CYP2D6,1.12261717959844e-05 |
| rs1968752 | Esophagus_Mucosa,SULT1A1,1.55283480376561e-11 |
| rs737980 | Esophagus_Gastroesophageal_Junction,THOC5,5.13357192148802e-06 |
| rs5743592 | Esophagus_Mucosa,TLR1,4.78432547034394e-13 |
| rs2239815 | Esophagus_Mucosa,HSCB,2.39841646435976e-05 |
| rs17002407 | Esophagus_Muscularis,CTA-223H9.9,2.22051956429303e-06 |
| rs2984526 | Esophagus_Muscularis,ZFAND5,3.91183755160585e-08 |
| rs3398 | Esophagus_Muscularis,ZFAND5,1.98214673870762e-07 |
