# Supplementary material for: Convergent evidence from systematic analysis of GWAS revealed genetic basis of esophageal cancer
Source: Oncotarget. 2016 Jun 17;7(28):44621–9. doi: 10.18632/oncotarget.10133 (PMC5190123; doi:10.18632/oncotarget.10133)
Supplement: Supplementary file 5 [file oncotarget-07-44621-s005.docx]

**Table S4** Details of pathway grouped network

| **Pathway groups** | **Group P-value** | **Included Pathway ID** | **Included Pathway title** |
| --- | --- | --- | --- |
| 1 | 8.26E-06 | GO:0042093 | T-helper cell differentiation |
|  |  | GO:0046631 | alpha-beta T cell activation |
|  |  | KEGG:05321 | Inflammatory bowel disease (IBD) |
|  |  | GO:0002377 | immunoglobulin production |
|  |  | GO:0002700 | regulation of production of molecular mediator of immune response |
|  |  | GO:0048634 | regulation of muscle organ development |
|  |  | GO:0048659 | smooth muscle cell proliferation |
|  |  | GO:0042307 | positive regulation of protein import into nucleus |
| 2 | 6.37E-03 | GO:0006656 | phosphatidylcholine biosynthetic process |
|  |  | GO:0042439 | ethanolamine-containing compound metabolic process |
| 3 | 1.51E-04 | GO:0042093 | T-helper cell differentiation |
|  |  | GO:0046631 | alpha-beta T cell activation |
|  |  | GO:0010883 | regulation of lipid storage |
|  |  | GO:0043368 | positive T cell selection |
|  |  | GO:0071354 | cellular response to interleukin-6 |
| 4 | 6.51E-06 | GO:0042093 | T-helper cell differentiation |
|  |  | KEGG:05321 | Inflammatory bowel disease (IBD) |
|  |  | GO:0002377 | immunoglobulin production |
|  |  | GO:0002700 | regulation of production of molecular mediator of immune response |
|  |  | GO:0048634 | regulation of muscle organ development |
|  |  | GO:0048659 | smooth muscle cell proliferation |
|  |  | GO:0032729 | positive regulation of interferon-gamma production |
|  |  | KEGG:05332 | Graft-versus-host disease |
|  |  | GO:0007431 | salivary gland development |
|  |  | GO:0031016 | pancreas development |
|  |  | GO:0031069 | hair follicle morphogenesis |
|  |  | GO:0033002 | muscle cell proliferation |
|  |  | GO:0034774 | secretory granule lumen |
|  |  | GO:0055025 | positive regulation of cardiac muscle tissue development |
|  |  | GO:0060038 | cardiac muscle cell proliferation |
|  |  | KEGG:05410 | Hypertrophic cardiomyopathy (HCM) |
| 5 | 3.96E-05 | GO:0005178 | integrin binding |
|  |  | GO:0050839 | cell adhesion molecule binding |
| 6 | 3.21E-08 | KEGG:05321 | Inflammatory bowel disease (IBD) |
|  |  | GO:0032729 | positive regulation of interferon-gamma production |
|  |  | KEGG:05332 | Graft-versus-host disease |
|  |  | GO:0030134 | ER to Golgi transport vesicle |
|  |  | GO:0030176 | integral component of endoplasmic reticulum membrane |
|  |  | GO:0060337 | type I interferon signaling pathway |
|  |  | GO:0071556 | integral component of lumenal side of endoplasmic reticulum membrane |
|  |  | GO:1902106 | negative regulation of leukocyte differentiation |
|  |  | KEGG:04514 | Cell adhesion molecules (CAMs) |
|  |  | KEGG:04940 | Type I diabetes mellitus |
|  |  | KEGG:05320 | Autoimmune thyroid disease |
|  |  | KEGG:05330 | Allograft rejection |
|  |  | KEGG:05416 | Viral myocarditis |
| 7 | 5.25E-03 | GO:0042987 | amyloid precursor protein catabolic process |
|  |  | GO:1901019 | regulation of calcium ion transmembrane transporter activity |
| 8 | 5.55E-03 | GO:0004022 | alcohol dehydrogenase (NAD) activity |
|  |  | GO:0004030 | aldehyde dehydrogenase [NAD(P)+] activity |
|  |  | GO:0005501 | retinoid binding |
|  |  | GO:0006069 | ethanol oxidation |
|  |  | GO:0006805 | xenobiotic metabolic process |
|  |  | GO:0046164 | alcohol catabolic process |
|  |  | KEGG:00010 | Glycolysis / Gluconeogenesis |
|  |  | KEGG:00071 | Fatty acid degradation |
|  |  | KEGG:00350 | Tyrosine metabolism |
|  |  | KEGG:00830 | Retinol metabolism |
|  |  | KEGG:00980 | Metabolism of xenobiotics by cytochrome P450 |
|  |  | KEGG:00982 | Drug metabolism |
|  |  | KEGG:05204 | Chemical carcinogenesis |
| 9 | 5.60E-03 | GO:0030818 | negative regulation of cAMP biosynthetic process |
|  |  | KEGG:05030 | Cocaine addiction |
| None | NA | GO:0006493 | protein O-linked glycosylation |
| None | NA | GO:0051965 | positive regulation of synapse assembly |
| None | NA | KEGG:00561 | Glycerolipid metabolism |

NA: Not applicable
